# Urine Parameters in Patients with COVID-19 Infection

**DOI:** 10.3390/life13081640

**Published:** 2023-07-28

**Authors:** Maria Morello, Dominga Amoroso, Felicia Losacco, Marco Viscovo, Massimo Pieri, Sergio Bernardini, Gaspare Adorno

**Affiliations:** 1 Clinical Biochemistry Department of Laboratory Medicine, Division of Proteins, University Hospital (PTV), 00133 Rome, Italy; felicialosacco@gmail.com (F.L.); marcoviscovo@outlook.it (M.V.); massimo.pieri@uniroma2.it (M.P.); bernards@uniroma2.it (S.B.); 2 Clinical Pathology and Clinical Biochemistry, Graduate School, Faculty of Medicine, University of Tor Vergata, 00133 Rome, Italy; gaspare.adorno@uniroma2.it; 3 Department of Experimental Medicine, Faculty of Medicine, University of Tor Vergata, 00133 Rome, Italy; 4 Department of Biomedicine and Prevention, University of Rome, 00133 Rome, Italy

**Keywords:** urinalysis, COVID-19 infection, urine parameters, biomarkers, inflammatory cytokines, acute kidney injury (AKI)

## Abstract

A urine test permits the measure of several urinary markers. This is a non-invasive method for early monitoring of potential kidney damage. In COVID-19 patients, alterations of urinary markers were observed. This review aims to evaluate the utility of urinalysis in predicting the severity of COVID-19. A total of 68 articles obtained from PubMed studies reported that (i) the severity of disease was related to haematuria and proteinuria and that (ii) typical alterations of the urinary sediment were noticed in COVID-19-associated AKI patients. This review emphasizes that urinalysis and microscopic examination support clinicians in diagnosing and predicting COVID-19 severity.

## 1. Introduction

COVID-19 (Coronavirus Disease 19) is an infection caused by the severe acute respiratory syndrome coronavirus 2 (SARS-CoV-2), and the disease was declared a pandemic by the World Health Organisation (WHO) on 11th March 2020 [1]. Patients with a SARS-CoV-2 infection may show different kinds of symptoms, from mild to severe ones [2]. Although COVID-19 is primarily an acute respiratory illness with nonspecific clinical symptoms associated with pneumonia involvement (such as flu, cough, dyspnea, myalgia, etc.), SARS-CoV-2 can involve multiple organs, such as the kidneys, heart, digestive tract, and nervous system. Precisely for this reason, the evaluation of the parameters derived from a routine laboratory test can indicate the involvement of the various organs. An interesting example is urinalysis, which can give an idea of the involvement of the kidneys in the disease. In fact, the involvement of the kidney in COVID-19 infection is reported since the virus can affect the glomeruli, tubules, or renal interstitium and can lead to acute kidney injury (AKI) [3,4,5]. Thus, urinary microscopy is an essential part of urinalysis that could be considered a valuable prognostic and diagnostic tool. The underlying mechanism of renal involvement is multifactorial and may be due to two main causes: (i) cellular damage induced by direct virus invasion into the renal parenchyma and/or (ii) a secondary effect due to systemic inflammation and sepsis-related cytokine storm. Severe inflammation of the kidney could contribute to hematuria and proteinuria [6].

In this review, the purpose is to summarize the “state of the art” concerning the association between urine biochemical parameters and COVID-19; in particular, we seek to understand which biomarkers help identify COVID-19 disease progression and its development to AKI, admission to the intensive care unit (ICU), and mortality. In addition, studies that reported results regarding microscopy of urinary sediment in COVID-19 patients have been analyzed in order to identify abnormalities, such as coarse granular casts (GCs), waxy casts (WxCs), and renal tubular epithelial cells (RTEC).

## 2. Methods: Identification and Characteristics of Included Studies

A total of 68 articles published between January 2020 and January 2023 were initially researched in the literature from PubMed using keywords: “COVID-19”, “urine parameters”, “inflammatory cytokines”, “urinalysis”. Of 68 articles, 27 articles (40%) reported a general description of COVID-19 patients without a distinction between different COVID-19 cases, and 41 articles (60%) provided a subdivision of COVID-19 patients in: moderate/severe COVID-19, COVID-19 AKI/non-AKI, and COVID-19 survivors and not survivors. In this review, we investigate if urine laboratory findings and inflammatory markers in COVID-19 patients are associated with worst outcome of disease. We studied 41 articles that included 21,996 COVID-19 patients; from these, 30 articles (73%) analyzed biochemical parameters, 6 articles (15%) reported urinary sediment, and 5 articles (12%) identified inflammatory cytokines in COVID-19 patients with different characteristics (Table 1).

### 2.1. Urine Biochemical Parameters for the Diagnosis and Prediction of COVID-19 Severity

**Figure 1:**
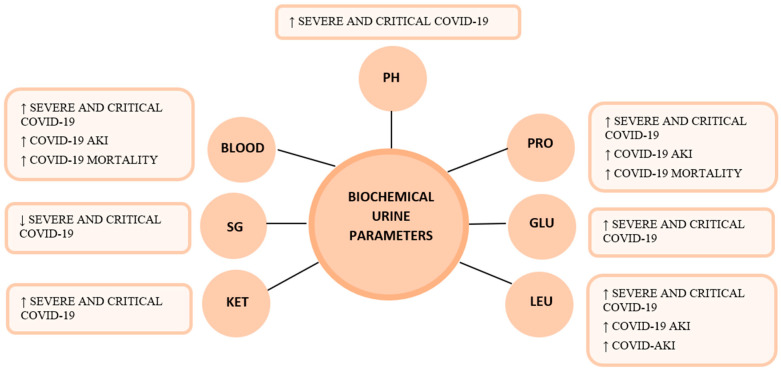
Biochemical urine parameters in COVID-19 patients, as reported in literature. Abbreviations: BLOOD, urine occult blood; PRO, proteinuria; GLU, glucose; LEU, leukocyturia; KET, ketones; SG, specific gravity; PH, potential of hydrogen. References: for PH [7,8,26,42]; for PRO (a) severe and critical COVID-19 [2,7,8,11,29], (b) COVID-19 AKI [25], (c) COVID-19 mortality [9,25]; for GLU [7,8,11]; for LEU (a) severe and critical COVID-19 [7,8,42], (b) COVID-19 AKI [25]; for KET [16]; for SG [4,5,10,16]; for BLOOD (a) severe and critical COVID-19 [7,8,10,11,29], (b) COVID-19 AKI [16,22,44], (c) COVID-19 mortality [14,16].

Specific urine indicators that are routinely quantified in a biochemistry laboratory are chosen to re-evaluate their utility in clinical practice (Table 2). Specifically, the urinalysis includes urine occult blood (BLOOD) that could be linked to glomerular damage, tumors, kidney damage, and kidney infections. Blood can also come transiently from menstruation and is associated with viral illnesses, strenuous exercise, and mild trauma. Some urinary metabolites will be analyzed for which the normal value is indicated in the literature as follows: red blood cells (RBC) in urine is between a concentration of 3 and 20 cells/uL; proteinuria (PRO) is used for the diagnosis of kidney disease and cardiovascular prevention and normal protein excretion is <150 mg/24 h; urinary glucose (GLU-U) represents the amount of ultrafiltered glucose, the normal concentration is ≤130 mg/dL, but this concentration could increase if the glucose concentration exceeds the tubular reabsorptive capacity (excessive glucose determination is typical in nephrological diseases such as tubulopathies and tubulointerstitial nephropathies); leukocyturia (LEU) is defined as the presence of 10–20 white blood cells (WBCs)/uL; a few WBCs can be found in normal centrifuged urine while pyuria generally indicates an infectious or inflammatory process in the kidney (pyelonephritis), bladder (cystitis), or urethra (urethritis); ketones (KET) are derived from fatty acid metabolism when dietary carbohydrate intake is low, and their presence in the urine has rarely a clinical utility except in certain situations, such as diabetic ketoacidosis and alcohol abuse; urine specific gravity (SG) is a function of the number and weight of dissolved particles indicating the ability of the kidney and its value varies from 1016 to 1022 g/mL; and pH reflects the regulation of the acid–base balance, its normal value varies from 5 to 8, and its measurement is important in urinary infections and is useful to evaluate tubular function [7,10,44]. Urine examinations in patients affected by COVID-19 were conducted in several studies and have been reported and summarized in this work (Figure 1). A series of observations was conducted by Liu, R et al. [7], Murgod, P. et al. [10], and Erdogan, O. et al. [8]. These studies involved patients with confirmed COVID-19 infection who were divided into three subgroups according to the severity of infection: “moderate”, “severe”, and “critical cases” and compared to healthy subjects (controls). The authors reported that BLOOD and PRO values were higher in COVID-19 patients than in control groups. In addition, when comparing COVID-19 patients to healthy control patients, other urinary parameters were altered, namely: SG and pH values were, respectively, found to be lower and higher in COVID-19 patients than in controls. These different results in COVID-19 patients were derived specifically from SARS-CoV-2 infection and not from other bacterial infections [7,8]. In addition, it was observed that patients of severe, and critical groups had values of GLU-U and PRO higher than patients of the moderate group. These results indicate that biochemical urine parameters are useful to show AKI in the advanced stages of the disease and could have a predictive role in disease severity [3,4,5,7,8,10,14,16]. The studies presented by Yildirim, C. et al. [9] and Bassoli, C et al. [11] evaluated PRO as an early predictor of COVID-19-related renal damage [9,11]. The study of Baulos et al. [42] and Sharma et al. [31] confirmed that abnormal urinalysis results, such as PRO, haematuria, and LEU were higher in severe COVID-19 infection and also in patients who died of the disease [29,31,42]. Observations from patients affected with the Omicron COVID-19 infection in the post-acute treatment phase were reported. The Omicron form is the less aggressive variant of SARS-CoV-2. In particular, a recent study by Teng and Chang that involved 409 patients (177 male and 232 female) affected by the Omicron form suggests that the incidence of haematuria and proteinuria is higher in patients with ordinary or mild symptoms (that are the majority of patients affected by the new Omicron variant). The cohort study involved patients that have been vaccinated before infection by COVID-19 as follows: one dose 20/409 (4.9%), two doses 204/409 (49.9%), and three doses 150/409 (36.7%). Based on the severity of clinical manifestation, patients were clinically grouped in asymptomatic 6/409 (1.5%), mild 201/409 (49.1%), ordinary 200/409 (48.9%), and severe 2/409 (0.5%). This study suggested that the majority of patients with Omicron infection showed ordinary and mild symptoms while the incidence of hematuria and proteinuria was, respectively, 14.7% and 14.2%, and concurrent hematuria and proteinuria were reported in 5.1% of the cases. Proteinuria and hematuria do not seem to be as clinically critical as AKI, but still, their role must be taken into account in the COVID-19 prognosis. Moreover, the study suggested that albuminuria and hematuria should be monitored throughout the clinical process of infection, including follow-up. The incidence of these two parameters was not as high as in the previously mentioned studies; however, it can predict adverse renal outcomes in long-term follow-up since Omicron infections may cause new kidney involvement [45].

### 2.2. Early Biomarkers of COVID-19-Related AKI

Many studies from the literature searches have tested the hypothesis that COVID-19 is correlated with the onset of AKI and the risk of death among patients with COVID-19 [19,20,36,46,47,48]. For this reason, various studies try to analyze urinary abnormalities in COVID-19 patients to identify predictors of AKI and mortality (Table 1). Depending on the pathophysiology of AKI and the duration of the COVID-19 disease, the incidence of AKI may vary. Especially in those patients requiring ICU support, AKI is associated with poor prognosis and increased risk of mortality. As a result of the studies mentioned above, we have observed that many laboratory parameters are useful as potential biomarkers to predict renal damage associated with COVID-19 [15,17,21,22,23,24,27,30,48,49,50].

**Figure 2:**
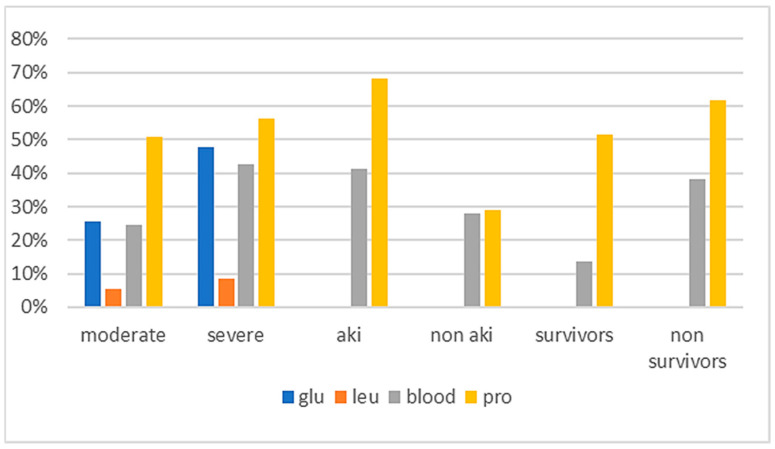
Graphic representation of the percentage of COVID-19 patients with different outcomes in whom principal biochemical parameters (GLU, LEU, BLOOD, PRO) are detected. BLOOD and PRO are correlated with worse outcomes of disease because they are higher in severe COVID-19 patients, AKI COVID-19 patients, and non-survivor patients.

A cytokine storm resulting from hyperinflammation and acute respiratory distress syndrome (ARDS) has also been reported to increase the frequency of AKI, which in turn increases the severity of COVID-19 [9]. As shown in Figure 2, all contributions that we included in this work highlighted that PRO was strongly associated with AKI [6,9,12,13,26]. To predict patient admission to the ICU as well as disease progression and mortality from COVID-19, Sundaram, S. et al. [12] and Morell-Garcia, D. et al. [13] observed an elevated presence of BLOOD in patients with AKI. In addition, the simultaneous increase in combined PRO and haematuria correlated with mortality in COVID-19 patients [12].

These data were confirmed by Morell-Garcia, D. et al. [13], who also noticed an increase in protein, glucose, ketones, and SG in severe cases of COVID-19 patients admitted to the ICU. By analyzing the impact of PRO on COVID-19 disease, the only prospective study mentioned in the review shows that higher urine protein level, ARDS, and nosocomial infection were significant factors for ICU admission [6]. Contrary to other results, this study found no differences in PRO, haematuria, LEU, and mortality among AKI patients compared with non-AKI patients; this discrepancy is probably due to the small number of subjects enrolled [6].

### 2.3. Role of the “Cytokine Storm” in COVID-19 Pathogenesis

COVID-19 is characterized by the activation of an inflammatory response defined as a cytokine storm, which derives from a dysregulated acquired immune system and hyperinflammatory innate immune responses. Cytokine storm is an elevated and uncontrolled production of pro-inflammatory cytokines that recruit inflammatory cells to the site of infection with destructive effects on human tissue with the destabilization of endothelial cells, vascular barrier damage, capillary damage, alveolar damage, kidney damage, multiorgan failure, and death [51].

**Figure 3:**
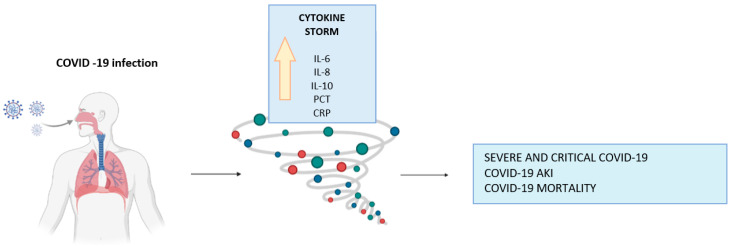
Association of inflammatory cytokines with different outcomes in hospitalized patients with COVID-19. Abbreviations: interleukin-6 (IL-6), interleukin-8 (IL-8), interleukin-10 (IL-10), procalcitonin level (PCT), C-reactive protein (CRP). References: SEVERE AND CRITICAL COVID-19 [44,54]; COVID-19 AKI [18,53]; COVID-19 MORTALITY [53].

Patients with severe COVID-19 and patients who died because of the disease showed elevated levels of inflammatory markers (Figure 3). These markers include interleukin-6 (IL-6), interleukin-8 (IL-8), interleukin-10 (IL-10), procalcitonin (PCT) level, and C-reactive protein (CRP) [52,53,54,55]. Some works highlight that COVID-19 patients admitted to ICU have higher serum levels of certain inflammatory cytokines, such as IL-6, CRP, and PCT, compared to other patients who are infected but less ill. Increased PRO and haematuria, linked to inflammatory markers, were also associated with AKI and mortality. The cytokine storm may contribute to AKI through cooperation with renal cells, and this causes tubular and endothelial dysfunction [18,39,40,53,56].

This evidence confirmed the involvement of the inflammatory cytokines related to disease severity and their correlations with urinary alterations [37,41,51]. Bacterial sepsis is a prototype to understand the pathogenesis of severe coronavirus disease; Patel, D.M. et al. [39] highlight that severe COVID-19 can be considered a sepsis syndrome caused by a viral infection as a consequence of various mechanisms including immune dysregulation, respiratory dysfunction leading to hypoxemia, and metabolic acidosis causing circulatory dysfunction [39]. Lin Y. et al. [4] emphasize that urine abnormalities, cytokine storms, and damage to the kidneys and other organs were similar to those seen in sepsis caused by bacterial infection, but there are also some differences. In COVID-19 sepsis, systemic inflammation is milder than in bacterial sepsis. Bacterial sepsis is characterized by an early-onset clinical deterioration while the viral disease has a late-onset and chronic course [4]. The study by Wilson, J.G. et al. [43] and Koçak Tufan, Z. et al. [57] suggests that a cytokine storm in severe COVID-19 was not markedly elevated when compared with critically ill patients with sepsis; this was seen through a “Luminex assay” that determined the expression of cytokines from the plasma of COVID-19 patients and patients with bacterial sepsis. These similarities and differences provide information about appropriate immunotherapy for SARS-CoV-2 sepsis [43,57].

### 2.4. Urinary Sediment Abnormalities in Patients with Renal Dysfunctions and COVID-19

Many studies support the implementation of urinary microscopy as an important diagnostic and prognostic procedure (Table 3). It has been revealed that the variability in the urinary sediment of patients with renal dysfunctions is commensurate with the clinical outcome in terms of renal function recovery, the need for dialysis and mortality [58]. The examination of urine sediment includes mainly cells, such as RBCs, WBCs, RTEC, and casts, that are classified as: hyaline, granular, waxy, fatty, and cellular (erythrocytic, leukocytic, and epithelial-containing RTEC casts). An increase in RBCs is associated with glomerulonephritis, lupus nephritis, tumors, acute infections, and toxic reactions to drugs; on the other hand, an increase in WBCs indicates the presence of an infection or inflammation of the urinary tract, and a high presence of RTEC reflects tubular injury. Urinary casts (tiny tube-shaped particles) have a morphology characterized by rounded and sometimes truncated ends and are formed by THP (Tamm-Horsfall protein), which may be the only constituent (as in hyaline cylinders) or by cellular or cell-derived elements. Casts derive from the aggregation of fibrils of THP, which is favoured by various factors, such as acid pH, high osmolality, and the presence of ultrafiltered proteins. Their presence in urinary sediment is useful to indicate renal failure and kidney diseases [59]. The study by Perazzella, M.A. et al. [60] shows that the frequency of granular casts and RTEC in the urine sediment is valuable to confirm the diagnosis of urinary tract disease and to differentiate acute tubular necrosis (ATN) from prerenal AKI [60]. The study by Pajenda, S. et al. [61] reveals that RTEC and glomerulus cells are the main targets of cytomegalovirus (CMV), leading to their cellular destruction and excretion in the urine. A CMV infection causes morbidity and mortality in kidney transplant patients [61]. Some publications show that WxCs in the urine sediment correlate well with renal disease, which had AKI and renal failure in common. These studies evaluated the presence of WxCs in different glomerular diseases. WxCs were found especially in acute postinfectious glomerulonephritis (GN) and renal amyloidosis; instead, they were absent in situations such as focal segmental glomerulosclerosis and rare in membranous nephropathy [61,62,63]. The study by Bagshaw S.M. et al. [63] reveals that urine sediment has an important clinical role in comparing urine sediment in septic AKI with non-septic AKI and in gaining insight into the severity of AKI in critically ill patients. It has been observed that more renal tubular cells and/or casts are present in the urinary sediment of septic patients than in non-septic AKI. Moreover, a greater kidney tubular injury has been observed in septic AKI than in non-septic AKI [63]. As reported in COVID-19 studies, urine abnormalities were also described in HIV-infected patients, and their presence was correlated with a severe disease outcome. The renal tubular injury, resulting in a significant decrease in glomerular function and an increase in the incidence of urinary protein, has been observed in HIV infection, and the same results were observed in COVID-19 patients. In COVID-19, such as in HIV, tubular protein excretions and glycosuria are markers of proximal tubular dysfunction and are useful for preventing cardiovascular disease risk and mortality in both HIV and COVID-19 patients [64,65]. An interesting work shows urinalysis abnormalities in a group of 226 COVID-19 patients hospitalized at the Valcamonica Hospital of Esine. In this group, 150 patients were male with a mean age of 66 years, and 72 were female with a mean age of 69 years. In patients who died, it was found an increased risk of haematuria (72.1%) and PRO (89.9%), and a more frequent presence of granular casts and tubular cells was observed in their urinary sediments. The higher risk of kidney failure in patients who died suggests that renal failure may be useful for mortality prediction in all patients with COVID-19 [33]. Microscopic examination of the urinary sediment (MicrExUrSed) in patients with COVID-19-associated AKI (CoV-AKI) could help in the diagnosis and prevention of AKI. Among 161 CoV-AKI patients, 20 patients (12%) underwent urinary sediment analysis. The vast majority of patients (85%, 17 out of 20) had only GCs or WxCs or RTEC casts in urinary sediment (Table 3). These patients also had significant LEU and haematuria [32,38].

Morell-Garcia et al. [13], who analyzed 199 COVID-19 patients to find urine biomarkers associated with AKI and mortality, confirmed an increased presence of pathological casts in the urinary sediments of the patients requiring ICU admission. MicrExUrSed is an effective clinical tool to predict AKI, which is a severe complication of COVID-19 and could help to optimize diagnostic interventions [25,34,66,67].

### 2.5. Prediction Tools for COVID-19 Detection Based on Urinalysis

In a multicentre cohort study, 223 COVID-19 patients admitted to four tertiary medical centers underwent urinalysis to predict the outcome of the disease. Among 223 COVID-19 patients, 145 patients were considered eligible for enrollment and were classified into three groups: patients with normal urinalysis (low-risk, *n* = 43); patients with abnormal urinalysis, serum albumin ≥ 2.0 g/dL, and AT-III activity ≥ 70% (intermediate-risk, *n* = 84); and patients with abnormal urinalysis, serum albumin < 2.0 g/dL, or AT-III activity < 70% (high-risk, *n* = 18). This study suggests that serum albumin and AT-III activity were particularly low among patients in the high-risk group. These markers were associated with worse outcomes from COVID-19. Low serum albumin induces fluid overload, interstitial pulmonary edema, and circulatory failure, which are the principal causes of death in COVID-19. Low AT-III activity causes thromboembolic events and counteracts the beneficial effect of heparin. This confirms that urine abnormalities may serve to build a prediction algorithm for COVID-19 severity detection [68].

Machine learning studies based on the use of algorithms highlight that biochemical, haematological, and urine biomarkers can be predictive factors for COVID-19 mortality [28]. The study by Gross, O. et al. [68] involved the use of four algorithms: artificial neural networks (ANN), decision trees (DT), discriminant analysis by partial least squares (PLS-DA), and the method of k-nearest neighbors (KNN). These models had 84–98% accuracy. Patients were divided into outpatients with non-severe diseases and hospitalized patients with severe diseases. The analysis showed hyperferritinemia, hypocalcemia, hypoxemia, pulmonary hypoxia, respiratory acidosis, metabolic acidosis, low urinary pH, and high levels of lactate dehydrogenase (LDH) in patients with severe disease [68]. Another review by Cobre, A. et al. [69], which included 189 studies (*n* = 57,563 patients), showed that patients with severe disease had significantly elevated levels of ferritin and low haemoglobin levels. These markers have been associated with the prediction of COVID-19 severity and are potential therapeutic targets [69].

Urine metabolism alterations were analyzed in urine samples of 102 healthy controls (HCs) and 248 COVID-19 patients using statistical approaches (PCA, PLS-DA, and OPLS-DA). This study showed that COVID-19 patients have significant alterations in urine metabolism, and in particular, 39 differential metabolites were responsible for discriminating between COVID-19 patients and HCs. A high proportion of urinary metabolites was from microbiota-associated metabolites (33.3%, 13/39) and the tryptophan metabolism pathway (12.8%, 5/39). This study indicates that urinary metabolites may be used for screening for COVID-19 and provide an understanding of microbiome composition in COVID-19 patients. Many researchers aim to build diagnostic models based on urine metabolites [35]. Recent studies are focused on how metabolomics analysis of COVID-19 patients can provide mechanistic information about the disease and can evaluate the long-term effect of such disease [70,71]. The study of Li et al. pointed out that urine proteomics analysis could be helpful in differentiating mild and severe COVID-19 patients. Changes in urine proteome and metabolic pathways will provide a better understanding of the pathogenesis of severe COVID-19 [70]. The implementation of NMR spectroscopy and LC-MS in the analysis of plasma samples made it possible to observe that metabolomic alterations caused by the COVID-19 infection and are partially restored after treatment with tocilizumab. Metabolic alterations were also present in vaccinated patients until three months after the first dose of vaccination in relation to the response of vaccination [72].

The study by Robertson et al. [71] shows that COVID-19 infection alters the molecular composition of urine and is pointed out by a metabolomic study. With the alterations in the profile of urinary metabolite, a sort of “fingerprint” can be determined by Raman spectroscopy and computational analysis. “Raman spectroscopy” is an inexpensive, rapid, and non-invasive technology that uses a chemometric approach. This new approach, compared to chromatographic and mass spectrometry technologies that resolve specific single molecules, is able to capture simultaneously hundreds of molecules in the urine. This study was possible due to the availability of a large dataset of urine “Raman spectra”. Urine Raman spectra of 46 symptomatic COVID-19 patients were compared with urine Raman spectra of healthy volunteers (*n* = 185), patients with chronic kidney disease (*n* = 20), and patients with bladder cancer (*n* = 17) collected between 2016–2018 (pre-COVID 19) and with urine from healthy vaccinated volunteers (*n* = 19) collected from July to September 2021. The results revealed: moderate (*n* = 25), mild (*n* = 14), and severe (*n* = 7) cases of COVID-19 and showed that Raman spectroscopy based on the urine multimolecular fingerprint is an accurate technology to identify mild, moderate, and severe symptoms. Considering that the use of different methods, their variability, and the changes associated with COVID-19 do not allow for a proper comparison between studies with statistical approaches [71], in order to better understand the urinalysis alteration, it is necessary to always have a complete clinical evaluation of the patients.

## 3. Conclusions

Clinical laboratory medicine is a crucial support in our reaction to COVID-19 because it helps limit intrinsic damage to tissue and organs and prevents worse outcomes. Researchers aim to implement new models based on urinalysis to predict disease behaviour and severity. These urinalysis findings from 41 articles that included 21,996 COVID-19 patients show that COVID-19 patients have significant urinary abnormalities, suggesting a worse disease progression and may be used for diagnosing and predicting COVID-19 severity. BLOOD and PRO especially are correlated with worse outcomes of disease because they are higher in severe COVID-19 patients (43%/56%), AKI COVID-19 patients (41%/68%), and non-survivor patients (38%/62%). Renal involvement may be a significant predictor of the disease progression toward severe cases, and it is possible to conclude that urinalysis may be performed in all patients with COVID-19. Urine microscopy and examination of the sediment have advantages because they are cheap, are readily available, and have technical simplicity with conventional equipment. Moreover, the examination of specific biochemical parameters derived from urine analysis, the correct manipulation of the obtained values, and the clinical observation of the symptoms can help us better understand the severity of the disease. The use of new computational techniques could, in fact, be of fundamental support, either for the prognosis of patients affected by severe COVID who can quickly develop AKI (hospitalized patients) or to monitor patients suffering from moderate COVID and/or vaccinated (not hospitalized) patients who could over time still have severe implications regarding kidney diseases.

## Figures and Tables

**Table 1:** Basic characteristics of included studies.

Analyzed Data	Ref.	Publication Date	Moderate and Mild COVID-19	Severe and Critical COVID-19	COVID-19 AKI	COVID-19 Non AKI	COVID-19 Survivors	COVID-19 Non-Survivors
Biochemical parameters	[7]	Jun 2020	67	52	undetected	undetected	undetected	undetected
	[8]	Nov 2021	85	48	undetected	undetected	undetected	undetected
	[9]	Jun 2021	undetected	undetected	17	331	undetected	undetected
	[10]	Aug 2021	230	55	undetected	undetected	undetected	undetected
	[11]	Apr 2021	130	77	undetected	undetected	undetected	undetected
	[12]	Feb 2021	undetected	undetected	undetected	undetected	83	27
	[13]	May 2021	undetected	undetected	15	184	179	20
	[6]	Mar 2021	23	22	undetected	undetected	undetected	undetected
	[3]	Oct 2022	undetected	444	undetected	undetected	undetected	undetected
	[4]	Nov 2022	undetected	1650	undetected	undetected	undetected	undetected
	[14]	Nov 2022	undetected	142	undetected	undetected	undetected	undetected
	[15]	Dec 2022	undetected	undetected	6	undetected	undetected	undetected
	[16]	Dec 2022	420	149	undetected	undetected	undetected	undetected
	[17]	Oct 2022	undetected	undetected	102	undetected	undetected	undetected
	[18]	Jan 2023	undetected	47	undetected	undetected	undetected	undetected
	[19]	Dec 2022	undetected	undetected	161	186	222	125
	[5]	Mar 2023	3946	715	undetected	undetected	3970	691
	[20]	Nov 2022	undetected	undetected	85	undetected	undetected	undetected
	[21]	Nov 2022	undetected	828	undetected	undetected	undetected	undetected
	[22]	Dec 2022	undetected	399	undetected	undetected	undetected	undetected
	[23]	Sep 2022	undetected	undetected	57	97	116	38
	[24]	Oct 2022	undetected	undetected	119	268	undetected	undetected
	[25]	Oct 2022	269	8	undetected	undetected	undetected	undetected
	[26]	Jun 2022	71	12	37	undetected	undetected	undetected
	[27]	Sep 2022	undetected	undetected	28	113	undetected	undetected
	[28]	Jun 2022	19	158	undetected	undetected	undetected	undetected
	[29]	Sep 2022	176	23	53	175	undetected	undetected
	[30]	Jan 2023	18	undetected	undetected	undetected	undetected	undetected
	[31]	Nov 2022	undetected	undetected	951	2059	undetected	undetected
	[32]	Apr 2022	undetected	undetected	27	50	undetected	undetected
Urinary sediment	[33]	Jun 2020	undetected	undetected	undetected	undetected	25	20
	[34]	Jun 2020	undetected	undetected	161	undetected	undetected	undetected
	[35]	Mar 2022	161	27	undetected	undetected	undetected	undetected
	[36]	Oct 2021	undetected	undetected	34	18	undetected	undetected
	[37]	Dec 2022	undetected	undetected	25	26	undetected	undetected
	[38]	Apr 2022	68	20	undetected	undetected	undetected	undetected
Inflammatory cytokines	[39]	May 2021	undetected	undetected	54	38	undetected	undetected
	[40]	Aug 2020	undetected	undetected	41	40	21	60
	[41]	Oct 2020	19	27	undetected	undetected	undetected	undetected
	[42]	Feb 2022	117	104	undetected	undetected	undetected	undetected
	[43]	Sep 2020	6	9	undetected	undetected	undetected	undetected

Number of patients in whom biochemical parameters, urinary sediment, and inflammatory cytokines are analyzed.

**Table 2:** Principal biochemical parameters (GLU, LEU, BLOOD, PRO) to predict COVID-19 progression and disease severity.

References	Biochemical Parameters	Partecipans of Studies	Moderate and Mild COVID-19	Severe and Critical COVID-19	COVID-19 AKI	COVID-19 Non AKI	COVID-19 Survivors	COVID-19 Non-Survivors	Results
		Patients Analyzed	372	155	63	594	262	47	
[7,8,10]	GLU	Patients with glycosuria	98	74	undetected	undetected	undetected	undetected	Glycosuria is higher in severe and critical COVID-19 patients (26.34%) than in moderat and mild COVID-19 patients (47.74%)
[7,8,10]	LEU	Patients with leukocituria	30	13	undetected	undetected	undetected	undetected	Leukocituria is higher in severe and critical COVID-19 patients (8.06%) than in moderate and mild COVID-19 patients (5.16%)
[7,8,9,10,12,13]	BLOOD	Patients with haematuria	94	66	26	166	36	18	Haematuria is higher in sever and critical COVID-19 (42.58%), in COVID-19 non survivors patients (38.30%) than in moderate and mild COVID-19 (25.27%), in COVID-19 non Aki (27.95%) and in COVID-19 survivors patients (13.74%).
[7,8,9,10,12,13]	PRO	Patients with proteinuria	194	87	43	172	135	29	Proteinuria is higher in severe and critical COVID-19 (56.13%), in COVID-19 AKI (68.25%), IN COVID-19 non survivors patients (61.70%) than in moderate and mild COVID-19 (52.15%), in COVID-19 non AKI (28.96%) and in COVID-19 survivors patients (51.53%).

**Table 3:** Microscopic examination of the urinary sediment. Abbreviations: GCs, coarse granular casts, WxCs (waxy casts), RTEC (renal tubular epithelial cells). Microscopic Images at 40×.

Renal Dysfunctions	Abnormalities in Urinary Sediment		
	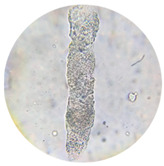	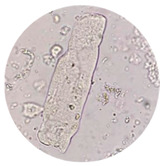	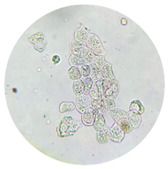
	GCs	WxCs	RTEC
Acute tubular necrosis	present	present	present
Post infectious glomerulonephritis	absent	absent	present
Renal amyloidosis	absent	absent	present
Focal segmental glomerulonephritis	absent	absent	absent
Nephropathy	absent	absent	rare
Septic AKI	present	present	present
COVID AKI patients	present	present	present
Death patients with COVID-19	high presence	high presence	high presence

## Data Availability

The datasets used were obtained after consultation of PubMed. The datasets generated during and/or analyzed during the current study are available from the authors on reasonable request.

## Abbreviations

Coronavirus Disease 19 (COVID-19); Severe Acute Respiratory Syndrome Coronavirus 2 (SARS-CoV-2); Acute Kidney Injury (AKI); Intensive Care Unit (ICU); Coarse granular casts (GCs); Waxy casts (WxCs); Renal tubular epithelial cells (RTEC); Urine occult blood (BLOOD); Red blood cells (RBC); Proteinuria (PRO); Urinary glucose (GLU-U); Leukocyturia (LEU); White blood cells (WBCs); Ketones (KET); Specific gravity (SG) Acute respiratory distress syndrome (ARDS); Interleukin-6 (IL-6); Interleukin-8 (IL-8); Interleukin-10 (IL-10); Procalcitonin (PCT); C-reactive protein (CRP); Tamm-Horsfall protein (THP); Acute Tubular Necrosis (ATN); Cytomegalovirus (CMV); Glomerulonephritis (GN); Microscopic examination of the urinary sediment (MicrExUrSed); Coronavirus disease 2019-associated AKI (CoV-AKI); Antithrombin III (AT-III); Healthy controls (HCs).
